# Advances in RNA Epigenetic Modifications in Hepatocellular Carcinoma and Potential Targeted Intervention Strategies

**DOI:** 10.3389/fcell.2021.777007

**Published:** 2021-10-29

**Authors:** Li-Ran Zhu, Wei-Jian Ni, Ming Cai, Wen-Tao Dai, Hong Zhou

**Affiliations:** ^1^Anhui Provincial Children’s Hospital, Anhui Institute of Pediatric Research, Hefei, China; ^2^The Key Laboratory of Anti-inflammatory of Immune Medicines, Inflammation and Immune Mediated Diseases Laboratory of Anhui Province, School of Pharmacy, Ministry of Education, Anhui Institute of Innovative Drugs, Anhui Medical University, Hefei, China; ^3^Anhui Provincial Hospital, The First Affiliated Hospital of USTC, Division of Life Sciences and Medicine, University of Science and Technology of China, Hefei, China; ^4^Department of Pharmacy, The Second Affiliated Hospital of Anhui University of Chinese Medicine, Hefei, China; ^5^School of Pharmacy, Anhui University of Chinese Medicine, Hefei, China; ^6^Key Laboratory of Chinese Medicinal Formula Research, Anhui University of Chinese Medicine, Hefei, China; ^7^Department of Pharmacy, Anhui Provincial Cancer Hospital, The First Affiliated Hospital of USTC, Division of Life Sciences and Medicine, University of Science and Technology of China, Hefei, China

**Keywords:** hepatocellular carcinoma, RNA epigenetic modification, N-methyladenosine, 5-methylcytosine, therapeutic targets

## Abstract

The current interventions for hepatocellular carcinoma (HCC) are not satisfactory, and more precise targets and promising strategies need to be explored. Recent research has demonstrated the non-negligible roles of RNA epigenetic modifications such as N6-methyladenosine (m6A) and 5-methylcytosine (m5C) in various cancers, including HCC. However, the specific targeting mechanisms are not well elucidated. In this review, we focus on the occurrence and detailed physiopathological roles of multiple RNA modifications on diverse RNAs closely related to the HCC process. In particular, we highlight fresh insights into the impact mechanisms of these posttranscriptional modifications on the whole progression of HCC. Furthermore, we analyzed the possibilities and significance of these modifications and regulators as potential therapeutic targets in HCC treatment, which provides the foundation for exploring targeted intervention strategies. This review will propel the identification of promising therapeutic targets and novel strategies that can be translated into clinical applications for HCC treatment.

## Introduction

According to the global cancer statistics report released by the International Agency for Research on Cancer (IARC), an estimated 18.1 million new cancer cases (17.0 million excluding non-melanoma skin cancer) and 9.6 million cancer deaths (9.5 million excluding non-melanoma skin cancer) occurred in 2018 worldwide (Bray et al., 2018). Among patients in 36 cancer types in 185 countries, liver cancer has become the sixth most commonly diagnosed cancer, with 840,000 new cases, and the cancer with the fourth highest mortality rate, with 782,000 deaths annually (de Martel et al., 2020).

Hepatocellular carcinoma (HCC) is mainly caused by chronic inflammatory liver diseases, such as viral liver disease and non-alcoholic and alcoholic fatty liver, and accounts for approximately 90% of primary liver cancers (PLCs) (Craig et al., 2020). Due to the asymptomatic nature of HCC in the early stage, most patients are diagnosed at an advanced stage, which results in limited therapeutic options and poor prognosis (Llovet et al., 2018). In the early stage of HCC, surgical resection, ablation therapy, and liver transplantation can be selected depending on the situation, but only 5–15% of patients are eligible (Hsu et al., 2021). Clinical studies have shown that transarterial chemoembolization (TACE) treatment can increase the 2-year survival rate of intermediate HCC patients by 23%; however, such choices are ineffective for advanced HCC patients (Raoul et al., 2019). In late-stage cases, one or multiple kinase inhibitors have to be used for chemotherapy, but only 1/3 of patients can benefit from this approach, and evident drug resistance is prone to develop within 6 months of initiating the regimen (Romero, 2020). Moreover, long-term use chemotherapeutic drugs, such as sorafenib and doxorubicin, will not only produce drug resistance but also become toxic and ineffective (Nwosu et al., 2020). In other words, neither surgery, ablation therapy nor chemotherapy can effectively improve the outcome of this devastating disease (Wang et al., 2021). There is an urgent need to further explore the pathogenesis of HCC and find effective treatments.

**FIGURE 1:**
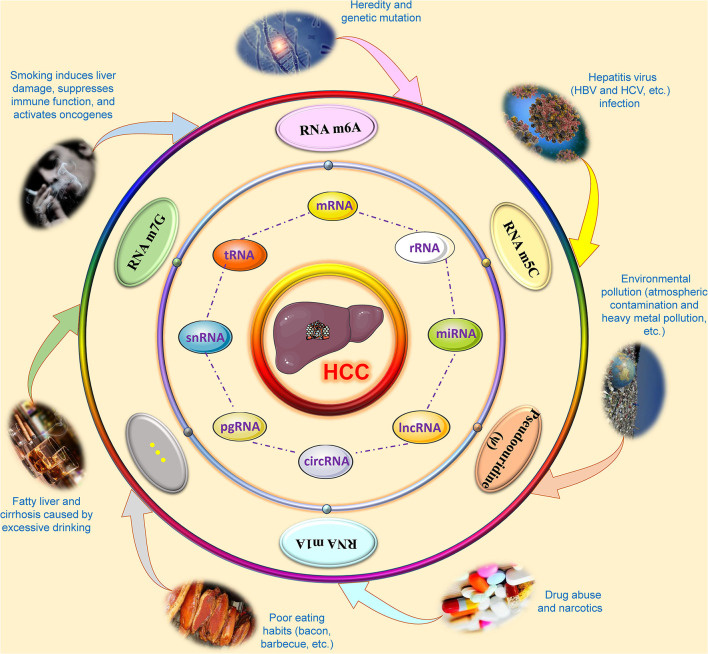
Overview of research on RNA epigenetic modifications of hepatocellular carcinoma. During the carcinogenesis and development of hepatocellular carcinoma, a variety of factors, such as smoking, alcohol, environmental pollution, viral infection, poor eating habits, drug abuse and narcotics may cause abnormal changes in epigenetic modifications in several kinds of RNAs based on genetic susceptibility, including N6-methyladenosine (m6A), 5-methylcytosine (m5C), N1-methyladenosine (m1A), N7-methylguanosine (m7G), and pseudouridine (ψ). These RNA epigenetic modifications will affect the metabolic and functional processes of RNA, such as RNA structure, splicing, stability, maturation, transport, translation, and degradation, by modifying the base or pyrimidine at a specific site of coding RNA and non-coding RNAs. These abnormalities in RNAs will affect the occurrence and progression of hepatocellular carcinoma through specific signal transduction or interactions. CircRNA, Circular RNA; HBV, Hepatitis B virus; HCC, Hepatocellular carcinoma; HCV, Hepatitis C virus; lncRNA, Long non-coding RNA; m1A, N1-methyladenosine; m5C, 5-methylcytosine; m6A, N6-methyladenosine; m7G, N7-methylguanosine; mRNA, Messenger RNA; miRNA, microRNA; pgRNA, pregenomic-RNA; rRNA, Ribosomal RNA; snRNA, Small nuclear RNA; tRNA, Transfer RNA.

Liver cancer is a complex malignant disease affected by multiple factors. Studies have confirmed that epigenetic modification can cause liver cancer by changing the expression of genes (Nagaraju et al., 2021). Etiological studies point out that a variety of environmental stresses lead to alterations in liver DNA methylation, acetylation, chromatin modification, and changes in non-coding RNAs, such as long non-coding RNAs (lncRNAs) and microRNAs (miRNAs), which will eventually cause changes in the liver epigenome and transcriptome, suggesting that epigenetic aberrations promote the initiation and promotion of HCC (Yang et al., 2019; Sinn et al., 2020). The accumulation of these epigenomic and epigenetic modifications and changes will cause dysfunction of antitumor genes and oncogenes, which are specifically manifested as carcinogenesis, development and metastasis of HCC (Huang et al., 2021). In other words, epigenetic modification provides a molecular supplement that can bridge the gap between genomic and environmental stresses, making the pathogenesis of HCC more complete. With the development of scientific research and technology, the exploration of the influence of RNA epigenetic modifications on the carcinogenesis and progression of HCC is ongoing, and some mechanisms have been elucidated. The overall outline is shown in Figure 1.

Recently, exploring the function of RNA modification in a variety of biological processes has become an emerging research hotspot (Zhao L. Y. et al., 2020). To date, more than 160 kinds of chemistry-related modification processes have been discovered, which are involved in the entire process of RNA function and metabolism (Helm and Motorin, 2017). Among them, the most common modifications currently include pseudouridine (ψ), N7-methylguanosine (m7G), 5-hydroxymethylcytosine (hm5C), N1-methyladenosine (m1A), 5-methylcytosine (m5C), and N6-methyladenosine (m6A) (Barbieri and Kouzarides, 2020). More recently, several authoritative studies have pointed out that multiple RNA (e.g., mRNA and ncRNA) epigenetic modifications and corresponding modifiers are involved in HCC cell proliferation, exacerbation and metastasis *via* epigenetic regulation of oncogenes and tumor suppressor genes, which urges us to explore and clarify the functions of RNA epigenetic modifications as soon as possible. Furthermore, targeting the modification process to develop novel drugs and technologies promotes knowledge regarding RNA-modifying processes and is an important therapeutic strategy for the development of specific therapies for HCC (Delaunay and Frye, 2019; Juhling et al., 2021).

Herein, the epigenetic situations and alterations of mRNA and ncRNAs, including mRNA, miRNAs, lncRNAs, and circular RNAs (circRNAs), in HCC are reviewed to summarize the epigenetic consequences of RNA modification in the pathogenesis of HCC, as the research focus and emerging direction in Table 1. In addition, we explored the role and possible mechanism of RNA epigenetic modification regulators to demonstrate their prospects as diagnostic markers and therapeutic targets for the treatment of HCC. Moreover, we also discussed the potential targeted intervention strategies for HCC based on the current research status of RNA epigenetic modification as well as questions to be addressed.

**TABLE 1:** A summary of known RNA epigenetic modifications and potential targets in HCC.

Modification type	Modification regulator	Gene name	Mechanism of action	References
**m6A**	METTL3	FOXO3 mRNA	METTL3 regulates sorafenib resistance in HCC through FOXO3-mediated autophagy	10.15252/embj.2019103181
	METTL3	SOCS2 mRNA	METTL3 promotes HCC progression through YTHDF2-dependent posttranscriptional silencing of SOCS2	10.1002/hep.29683
	METTL3	LINC00958	METTL3-mediated upregulation of LINC00958 increases lipogenesis in HCC	10.1186/s13045-019-0839-x
	METTL3	FEN1 mRNA	METTL3 and IGF2BP2 maintained FEN1 expression through an m6A-IGF2BP2-dependent mechanism in HCC	10.3389/fonc.2020.578816
	METTL3	Snail mRNA	SUMO1 modification of METTL3 promotes tumor progression *via* regulating Snail mRNA homeostasis in HCC	10.7150/thno.42539
	METTL3	circ-ARL3	METTL3 promotes circ-ARL3 to facilitate HBV-associated HCC *via* sponging miR-1305	10.1002/iub.2438
	METTL3	CD47 mRNA	METTL3/IGF2BP1/CD47 mediated EMTcontributes to the incomplete ablation induced metastasis in HCC cells	10.1016/j.bbrc.2021.01.085
	METTL3	RDM1 mRNA	METTL3 represses RDM1 to increase cell proliferation, colony formation in HCC	10.1002/1878-0261.12593
	METTL3	lncRNA NIFK-AS1	METTL3 upregulates lncRNA NIFK-AS1 to promotes HCC progression and sorafenib resistance	10.1007/s13577-021-00587-z
	METTL3	LncRNA MEG3	METTL3-induced LncRNA MEG3 to suppress the proliferation, migration and invasion of HCC through miR-544b/BTG2 signaling	10.2147/OTT.S289198
	METTL14	EGFR mRNA	METTL14 inhibits HCC Metastasis through regulating EGFR/PI3K/Akt signaling	10.2147/CMAR.S286275
	METTL14	HNF3γ mRNA	METTL14-mediated HNF3γ reduction renders HCC dedifferentiation and sorafenib resistance	10.1038/s41392-020-00299-0
	METTL14	USP48 mRNA	METTL14 upregulates USP48 to attenuate HCC *via* regulating SIRT6 stabilization	10.1158/0008-5472.CAN-20-4163
	WTAP	ETS1 mRNA	WTAP facilitates progression of HCC *via* m6A-HuR-dependent epigenetic silencing of ETS1	10.1186/s12943-019-1053-8
	VIRMA	GATA3 mRNA	VIRMA contributes to HCC progression through m6A-dependent post-transcriptional modification of GATA3	10.1186/s12943-019-1106-z
	FTO	CUL4A mRNA	FTO reduces CUL4A abundance to reverse the increased hepatocyte proliferation in HCC	10.1016/j.molmet.2020.101085
	FTO	PKM2 mRNA	FTO promotes HCC tumorigenesis *via* mediating PKM2 demethylation	PMID: 31632576; PMID: PMC6789218
	FTO	GNAO1 mRNA	SIRT1 destabilizes FTO to steering the GNAO1 mRNA expression in HCC tumorigenesis	10.1002/hep.31222
	ALKBH5	LYPD1 mRNA	ALKBH5 suppresses malignancy of HCC *via* m6A-guided epigenetic inhibition of LYPD1	10.1186/s12943-020-01239-w
	ALKBH5	HBx mRNA	ALKBH5 catalyzes HBx mRNA to promote HBV-driven HCC cells’ growth and migration	10.1186/s12885-021-08449-5
	YTHDF1	ATG2A and ATG14 mRNA	YTHDF1 drives hypoxia-induced autophagy and malignancy of HCC by promoting ATG2A and ATG14 translation	10.1038/s41392-020-00453-8
	YTHDF1	Akt mRNA	YTHDF1 promotes HCC progression *via* activating PI3K/AKT/mTOR signaling and inducing EMT	10.1186/s40164-021-00227-0
	YTHDF1	FZD5 mRNA	YTHDF1 facilitates the progression of HCC by promoting FZD5 mRNA translation	10.1016/j.omtn.2020.09.036
	YTHDF1	Akt mRNA	YTHDF1 promote the HCC cell aggressive phenotypes by facilitating EMT and activating Akt/glycogen GSK-3β/β-catenin signaling	10.3389/fmolb.2020.60476
	YTHDF2	IL11 and SERPINE2 mRNA	YTHDF2 processed the decay of IL11 and SERPINE2 mRNAs responsible for the inflammation-mediated malignancy and disruption of vascular normalization in HCC	10.1186/s12943-019-1082-3
	YTHDF2	OCT4 mRNA	YTHDF2 promotes the liver cancer stem cell phenotype and cancer metastasis by regulating OCT4 expression	10.1038/s41388-020-1303-7
	YTHDF2	EGFR mRNA	YTHDF2 suppresses cell proliferation and growth *via* destabilizing the EGFR mRNA in HCC	10.1016/j.canlet.2018.11.006
	YTHDF3	Zeb1 mRNA	circ_KIAA1429 accelerates HCC advancement through m6A-YTHDF3-Zeb1	10.1016/j.lfs.2020.118082
	YTHDF3	ITGA6 mRNA	KDM5B promotes self-renewal of HCC cells through the microRNA-448-mediated YTHDF3/ITGA6 axis	10.1111/jcmm.16342
**m5C**	NSUN2	H19 lncRNA	Aberrant NSUN2-mediated m(5)C modification of H19 lncRNA is associated with poor differentiation of HCC	10.1038/s41388-020-01475-w
	NSUN2	FZR1 mRNA	NSUN2 promotes growth of HCC cells by regulating FZR1 *in vitro* and *in vivo*	10.1002/kjm2.12430
	TET1/TET2	SOCS1 mRNA	miR-29a down-regulates anti-metastatic SOCS1 by directly targeting the TET family	10.1007/s12032-014-0291-2
**m7G**	METTL1	PTEN mRNA	METTL1 overexpression is correlated with poor prognosis and promotes HCC *via* PTEN	10.1007/s00109-019-01830-9
	WDR4	CCNB1 mRNA	WDR4 promotes proliferation, metastasis, and sorafenib resistance by inducing CCNB1 translation in HCC	10.1038/s41419-021-03973-5
**Pseudouridine**	Unknown	18S rRNA component of the small (40S) ribosomal subunit	snoRNA24 guided pseudouridine modifications increased translational miscoding and stop codon readthrough frequencies in HCC	10.7554/eLife.48847

*ALKBH5, AlkB homolog 5; ATG2A, Autophagy related 2a; BTG2, B cell translocation gene 2; CCNB1, cyclin B1; CUL4A, Cullin 4A; EGFR, Epidermal growth factor receptor; EMT, Epithelial-to-mesenchymal transition; ETS1, ETS proto-oncogene 1; FEN1, Flap endonuclease-1; FOXO3, Forkhead transcription factor 3; FTO, Fat mass and obesity-related protein; FZD5, Frizzled homolog 5; FZR1, Fizzy-related-1; GATA3, GATA binding protein 3; GNAO1, Guanine nucleotide-binding protein, α-activating activity polypeptide O; HBV, Hepatitis B virus; HBx, HBV derived X protein; HCC, hepatocellular carcinoma; HNF3γ, Hepatocyte nuclear factor 3γ; IGF2BP1, Insulin-like growth factor 2 mRNA binding protein 1; IL11, Interleukin 11; ITGA6, integrin subunit alpha 6; KDM5B, Lysine-specific demethylase 5B; LYPD1, LY6/PLAUR domain containing 1; m5C, 5-methylcytosine; m6A, N6-methyladenosine; m7G, N7-methylguanosine; METTL14, Methyltransferase-like 14; METTL3, Methyltransferase-like 3; NSUN2, NOP2/Sun domain family, member 2; OCT4, Octamer-binding transcription factor 4; PKM2, Pyruvate kinase type M2; PTEN, Phosphatase and tensin homolog; RDM1, RAD52 motif 1; SERPINE2, Serpin family E member 2; SIRT1/6, Silent information regulator 1/6; SNORA24, Small nucleolar RNA 24; SOCS1/2, Suppressor of cytokine signaling 1/2; SUMO1, Small ubiquitin-like modifier; TET1/TET2, Ten-eleven translocation methyl-cytosine dioxygenase 1/2; USP48, Ubiquitin-specific peptidase 48; VIRMA, Vir-like m6A methyltransferase with association; WDR4, WD repeat domain 4; WTAP, Wilms tumor 1-associating protein 1.*

## RNA N6-Methyladenosine Epigenetic Modification in Hepatocellular Carcinoma

The m6A modification of RNA presents a methylating process in the adenosine N6 site, which is the most common and abundant RNA methylation modification and was found in > 25% of mRNAs in eukaryotes (Zhang H. et al., 2020). A number of studies have found that m6A methylation modification regulates multiple metabolic processes of RNA, including RNA structure, splicing, stability, maturation, transport, translation, and degradation (Xiao et al., 2019; Zhang et al., 2021). Meanwhile, authoritative studies have proposed that m6A methylation modification not only exists in the metabolic process of mRNA but can also affect the biological functions of non-coding RNA, and the impact of this modification cannot be ignored (Niu et al., 2013; Roundtree et al., 2017). Briefly, the process of RNA m6A methylation is mainly catalyzed by a complex methyltransferase complex, methyltransferase-like 14 (METTL14) and methyltransferase-like 3 (METTL3). During this catalytic process, several other protein subunits are required to participate, mainly consisting of motif protein 15/15B (RBM15/15B) that binds to RNA, Vir-like m6A methyltransferase with association (VIRMA), zinc finger CCCH-type covering 13 (ZC3H13), and Wilms tumor 1-associating protein (WTAP) (Liu et al., 2014; Abakir et al., 2020). When RNAs undergo m6A methylation, they may also experience reversible demethylation, which is mainly regulated by two enzymes, fat mass and the obesity-related protein (FTO) and human AlkB homolog 5 (ALKBH5), i.e., the so-called “eraser.” In addition, m6A-modifying processes that cause structural alterations of RNA can be identified by a selective RNA binding protein, namely, m6A reading elements (Meyer and Jaffrey, 2017). At present, the major known reading elements include the protein family consisting of the YT521-B homology (YTH) domain [e.g., the Homo sapiens nuclear YTH domain containing 1 (YTHDC1) and the cytoplasmic YTH domains YTHDF1, YTHDF2, YTHDF3, and YTHDC2] (Zaccara and Jaffrey, 2020). With the deepening of research, some previously unknown RNA binding proteins, such as nuclear factor κB (NF-κB)-related protein (NKAP) and heterogeneous nuclear protein (HNRNP) families (HNRNPA2B1, HNRNPG, and HNRNPC), have also been identified *via* particular m6A identification to be able to assist the m6A process to affect the RNAs’ fate as well as their functions exhibited by cells (Zaccara et al., 2019). Increasing evidence shows that the total m6A level and its regulators in HCC tissue have abnormal changes and are closely related to poor clinical prognosis (Lu et al., 2021; Xu et al., 2021). Furthermore, m6A-modified regulators exert a carcinogenic or antitumor effect in HCC by affecting the expression of certain specific genes (Li et al., 2021). Therefore, clarifying the role and specific mechanism of RNA m6A modification in HCC, finding promising therapeutic targets and developing new targeted intervention strategies will greatly promote the treatment of HCC in the future.

**FIGURE 2:**
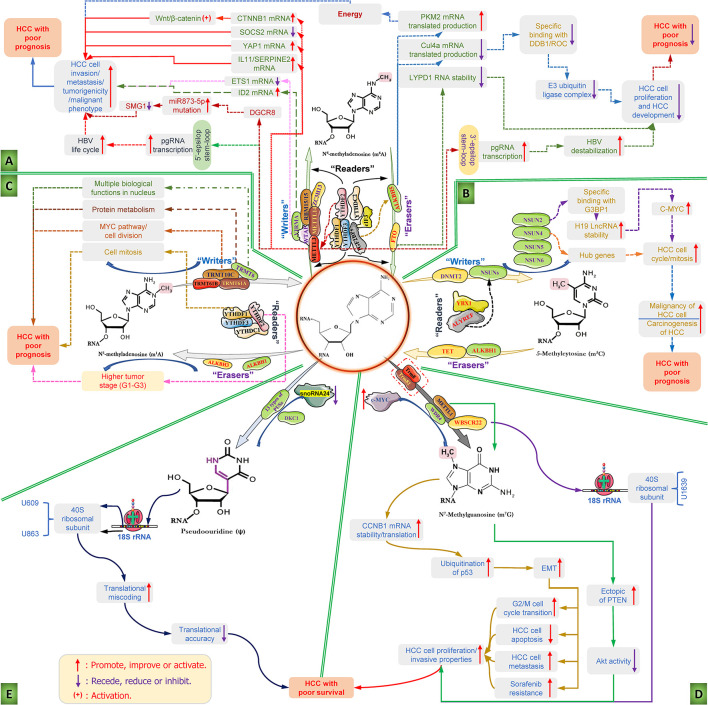
Diverse RNA epigenetic modifications and their functions in the carcinogenesis and progression of hepatocellular carcinoma. RNA epigenetic modifications, including N6-methyladenosine (m6A), 5 methylcytosine (m5C), N1-methyladenosine (m1A), N7-methylguanosine (m7G), and pseudouridine (ψ), have been reported to play major roles in the invasion, metastasis and malignant transformation of HCC cells and the progression of HCC. **(A)** The mechanism diagram mainly illustrates the roles of the dysfunction of RNA m6A modification regulators and the corresponding m6A modifications regulates the key influencing factors and the whole course of HCC development; **(B)** the detailed roles of RNA m5C modification regulators dysfunction and the corresponding m5C modifications regulates the carcinogenesis and progression of HCC; **(C)** deregulation of m1A modifiers and the related RNA m1A modifications in the carcinogenesis and progression of HCC; **(D)** deregulation of m7G modifiers and the related RNA m7G modifications in the development features of liver cancer cell and carcinogenesis and progression of HCC; and **(E)** role of pseudouridine (ψ)-mediated pseudouridylation of 18S rRNA in the HCC with poor survival. ALKBH1/3/5, AlkB homolog 1/3/5; ALYREF, Aly/REF export factor; C-MYC, MYC proto-oncogene; CTNNB1, catenin (cadherin-associated protein), beta 1; CUL4A, Cullin 4A; DDB1, DNA damage binding protein 1; DGCR8, DiGeorge critical region 8; DKC1, Dyskerin pseudouridine synthase 1; DNMT2, DNA methyltransferase; elF3, E74-like factor 3; EMT, Epithelial-to-mesenchymal transition; ETS1, ETS proto-oncogene 1; FTO, fat mass and obesity-related protein; G3BP1, Ras GTPase-activating protein-binding protein 1; HBV, hepatitis B virus; HCC, hepatocellular carcinoma; HCV, hepatitis C virus; IGF2BP1/2/3, insulin like growth factor 2 mRNA binding protein 1/2/3; IL11, interleukin 11; lncRNA, long non-coding RNA; LYPD1, LY6/PLAUR Domain Containing 1; m1A, N1-methyladenosine; m5C, 5-methylcytosine; m6A, N6-methyladenosine; m7G, N7-methylguanosine; METTL3/14, methyltransferase-like 3/14; NSUN2/4/5/6, NOP2/Sun domain family, member 2/4/5/6; pgRNA, pregenomic RNA; ψ, pseudouridine; PKM2, Pyruvate kinase type M2; PTEN, Phosphatase and tensin homolog; RBM15/15B, RNA binding motif protein 15/15B; SERPINE2, serpin family E member 2; SMG1, suppressor with the morphological effect on genitalia 1; snoRNA24, Small nucleolar RNA 24; SOCS2, suppressor of cytokine signaling 2; TET, Ten-eleven translocation methyl-cytosine dioxygenase; TRMT, Transfer RNA methyltransferase; TRUB1, TruB PSU class members 1; VIRMA, Vir-like m6A methyltransferase with association; WBSCR22, Williams-Beuren syndrome chromosome region 22; WDR4, WD repeat domain 4; WTAP, Wilms tumor 1-associating protein; YAP1, Yes1 associated transcriptional regulator; YBX1, Y-box binding protein 1; YTHDC, YTH domain-containing reader protein; YTHDF, YT521-B homology domain family; ZC3H13, zinc finger CCCH-type covering 13.

At present, there have been many studies on the role and possible mechanism of RNA m6A modification and the corresponding regulatory factors in HCC, as shown in Figure 2A. In the early stages of this disease, a study showed that a high-fat diet can induce elevated m6A modification in cancer tissues and is partially enriched in lipid metabolism-related genes and processes, including cell lipid metabolism, fatty acid metabolism, and triglyceride metabolic reactions, which are among the most critical risk factors for hepatocarcinogenesis (Luo et al., 2019). A study showed that flavivirus infection can affect the m6A methylation modification of liver tissue, thereby changing the m6A level in special transcripts and, in turn, promoting viral infections (Gokhale et al., 2020). Further research found that METTL3/14-mediated m6A modification in the 5′-epsilon stem-loop of pregenomic RNA (pgRNA) is necessary for effective reverse transcription of pgRNA, thus mediating the life cycle of hepatitis B virus (HBV), while m6A modifications in the 3′-epsilon stem-loop lead to the destabilization of HBV transcripts and have an antiviral effect (Imam et al., 2018). The results of this study indicate that m6A modification at different sites of HBV RNA has completely different effects, which will provide an important targeted intervention strategy for antiviral therapy of HCC. Hepatitis C virus (HCV) infection is an important risk factor for HCC. A study showed that there are signals of m6A methylation modification present in the RNA genome of HCV, and these signals can be regulated by the YTHDF reading protein. YTHDF proteins reduce the production of HCV particles through the cellular m6A machinery and are located at the site of virus assembly and intracellular lipid droplets, thereby reducing HCV infection and the occurrence of HCC (Gokhale et al., 2016). Adding m6A-abrogating mutations within HCV RNA or depleting the cellular m6A machinery can increase HCV particle production, infection and even the risk of HCC, suggesting that m6A negatively regulates HCV. The above study suggests that the regulation of m6A modification can be used as a potential intervention strategy for HCC induced by HCV infection. Angiogenesis is one of the essential factors in the progression and metastasis of HCC. A study found that m6A modification can affect the progression of HCC by controlling angiogenesis. In this study, researchers found that silencing METTL3 inhibits the expression of yes-associated protein 1 (YAP1) and reduce the formation of angiogenic mimicry (Qiao et al., 2021). Moreover, silencing the reading protein YTHDF2 reduces the degradation of two m6A-modified mRNAs, including interleukin 11 (IL11) and serpin family E member 2 (SERPINE2), which, in turn, escalates the abnormalization of vessels and inflammation and eventually promotes HCC growth, vasculature remodeling and metastasis (Hou et al., 2019). These studies indicate that different regulator-mediated m6A modifications affect HCC progression and metastasis in the early stages of HCC, suggesting that these regulator proteins may be considered potential research and therapeutic targets, and influencing m6A modification by regulating these targets is a promising targeted intervention strategy for HCC treatment.

During HCC progression, METTL3 promotes the expression of Snail, a key transcription factor that regulates epithelial-mesenchymal transition (EMT), through YTHDF1-dependent m6A modification, thereby facilitating the migration, invasion and deterioration of cancer cells (Lin X. et al., 2019). Furthermore, the ubiquitin-like modifier SUMO1, which mediates the ubiquitination of METTL3, further promotes these processes and is highly positively correlated with the high metastatic potential of HCC (Xu et al., 2020). In addition, METTL3 was found to accelerate the suppressor of cytokine signaling 2 (SOCS2) mRNA decay *via* YTHDF2-dependent m6A modification and reduce the level of SOCS2 in malignant tissue, thereby abrogating the inhibitory effect of SOCS2 on liver cancer cell proliferation, migration and stem cell characteristics (Chen et al., 2018). In addition, a study also found that METTL3, which is significantly upregulated in hepatoblastoma (HB), can enhance the stability of catenin (cadherin-associated protein) beta 1 (CTNNB1) mRNA in a m6A modification-dependent manner and, subsequently, activate Wnt/β-catenin signaling to expedite HB progression (Liu L. et al., 2019). In addition to focusing on the function of the METTL3/14 complex, other methyltransferases have also been reported to play a pivotal role during HCC progression. Among them, WTAP leads to the posttranscriptional suppression of ETS proto-oncogene 1 (ETS1) in a m6A-dependent manner by using Hu-Antigen R (HuR) as an RNA stabilizer, thus regulating the HCC cell cycle distribution of G2/M phase in a p21/p27-dependent manner to speed up HCC progression (Chen et al., 2019). In addition, one study also found that VIRMA, also known as KIAA1492, is highly expressed in HCC and inhibits the level of ID2 mRNA through m6A modification, promoting the metastasis and invasion of HCC (Cheng et al., 2019). In addition to regulating the mRNA m6A modification, METTL3 has been reported to interact with the DiGeorge critical region 8 (DGCR8) by a m6A modification pattern to boost the maturation of miRNA-873-5p and inhibit the expression of the suppressor with the morphological effect on genitalia 1 (SMG1), thus enhancing the tumorigenicity and malignant phenotype of liver cancer cells (Zhao M. et al., 2020). Additionally, METTL3-mediated m6A modification can increase the stability of the lncR00958 transcript and, thus, upregulate lnc00958 expression. Subsequently, lncR00958 elevated the level of hepatoma-derived growth factor (HDGF) by recruiting miR-3619-5p and ultimately promoted HCC lipogenesis and progression (Zuo et al., 2020). In addition, researchers have sought to identify abnormally changed circRNAs closely related to KIAA1492 in HCC tissues and further study their functions. The results show that KIAA1492-mediated hsa-circ_0084922 alteration can enhance the stability of Zeb1 mRNA in a m6A-YTHDF3-dependent manner, thereby facilitating the migration, EMT and invasion of HCC cells and accelerating HCC progression (Wang et al., 2020). A large number of research results show that the “writers” and “readers” of m6A modification play essential roles in the occurrence and development of HCC, which provides a pivotal cornerstone for exploring novel targets for HCC treatment. Moreover, targeting writer and reader targets to regulate m6A modification provides a potential targeted strategy for future HCC treatment.

Since FTO was identified as a m6A demethylase, it has been reported to be involved in the regulation of a variety of cancers, including HCC. A study showed that FTO triggered the demethylation of pyruvate kinase type M2 (PKM2) to accelerate its translated production and promote HCC oncogenesis by providing it with energy. In addition to its oncogenic function, FTO also suppresses HCC progression (Li et al., 2019). In DEN-induced HCC using mice with hepatic FTO deficiency, abundant m6A modifications were observed, which promotes the translation of Cullin 4a (Cul4a) mRNA to increase its protein level. The elevated scaffold protein Cul4a binds DNA damage binding protein 1 (DDB1) and the ring of cullins (ROC) to assemble an E3 ubiquitin ligase complex, which positively correlates with hepatocyte proliferation, HCC development and progression (Mittenbuhler et al., 2020). In addition, ALKBH5 was reported to inhibit the proliferation and invasion of HCC cells by decreasing insulin-like growth factor 2 mRNA-binding protein 1 (IGF2BP1)-mediated LY6/PLAUR domain containing 1 (LYPD1) RNA stability (Chen et al., 2020). The above studies suggest that demethylase-mediated m6A modification seems to play a “positive energy” role in the progression of HCC. Therefore, these enzymes, including FTO and ALKBH5, should be further studied to evaluate their potential as therapeutic targets and targeted intervention strategies based on these enzymes in HCC treatment.

The carcinogenic and tumor suppressor effects of m6A readers are mainly related to their impact on transcript processing. Ding et al. (2020) found that YTHDF1-dependent m6A modification can enhance the expression of LG-protein alpha-subunit (GNAS) to promote STAT3 activation by preventing the interaction of lncRNA TPTEP1 and STAT3 (signal transducer and activator of transcription 3), thus accelerating inflammation-related HCC progression in LPS-stimulated HCC cells (Nault et al., 2012). YTHDF2 has been proven to mediate the liver cancer stem cell phenotype and tumor metastasis by promoting the expression of OCT4 (Zhang C. et al., 2020). Moreover, inhibiting the expression of YTHDF2 can increase the m6A level of IL11 and SERPINE2 mRNA, thus aggravating inflammation and vascular abnormalities and ultimately promoting the development of HCC (Hou et al., 2019). Moreover, IGF2BP1/2/3 has been proven to be a m6A modification binding protein that exerts oncogenic effects in liver cancer cells by enhancing the stability of MYC, FSCN1, and TK1 mRNA (Huang et al., 2018). In summary, multiple m6A regulators and the corresponding m6A modifications play a variety of roles in the carcinogenesis and progression of HCC, specifically manifested in regulating HCC cell phenotype, vascular abnormalities, migration, invasion, and EMT processes. Although there are conflicting results in some studies, this does not affect the m6A “writers,” “erasers,” and “readers” as potential therapeutic targets for the treatment of HCC, nor does it affect the development of targeted intervention strategies based on m6A modifications. Although there have been great developments in the study of m6A modification in liver cancer, there is still a long way to go before truly elucidating the role and mechanism of m6A modification in HCC, as well as the confirmation and clinical application of targeted therapy strategies. Therefore, further investigation and efforts are needed to promote the smooth progress of this work.

## RNA 5-Methylcytosine Epigenetic Modification in Hepatocellular Carcinoma

M5C is another posttranscriptional RNA modification mode that has been confirmed by multiple studies to have an important regulatory role in RNA metabolism (Breiling and Lyko, 2015; Li et al., 2019). Initial studies have shown that m5C mainly exists in ribosomal RNA and transfer RNA, but recently, it was found that m5C is significantly expressed in mRNA as well, which is catalyzed by members of the NOP2/Sun RNA methyltransferase family (Van Haute et al., 2019; Shi H. et al., 2020). Combined with a series of authoritative studies, the RNA m5C modification is mainly catalyzed by the NOL1/NOP2/SUN domain (NSUN) domain protein family, including NSUN1, 2, 3, 4, 5, 6, and 7, and DNA methyltransferase member 2 [DNMT2 or TRNA Aspartic Acid Methyltransferase 1(TRDMT1)] (Khoddami et al., 2019). Studies have found that NSUN2 methylates the majority of tRNAs at the variable loop location, while DNMT2 methylates the anti-codon loop of three tRNAs at the wobble position of leucine, and NSUN6 targets a few acceptor stems of tRNAs. NSUN4 mainly targets the small subunit of rRNA in mitochondria, while NSUN3 is responsible for the tRNAs of mitochondria, which are essential for 5-formylcytosine formation. In addition, NSUN1 (NOP2) and NSUN5 are very conserved residues in the nucleolus and methylation of 28S rRNA, which are located near the center of peptidase and the interface between large and small subunits, respectively (Huang et al., 2019). During this process, Y-box binding protein 1 (YBX1) and Aly/REF export factor (ALYREF) were considered m5C readers, which play a critical role in RNA metabolic processes, such as RNA processing, stability, export, and RNA translation (Khoddami et al., 2019). In regard to demethylation, studies have confirmed that m5C in mRNA can be oxidized by the ten–eleven translocation protein (TET) enzyme family to produce 5-hydroxymethylcytosine (hm5C) or form f5C by the α-ketoglutarate-dependent dioxygenase ALKBH1 in tRNAs of the mitochondria (Chen et al., 2021). The m5C modification in the mammalian transcriptome is highly conserved, tissue-specific, and dynamic. However, many studies have found that aberrant m5C modification may lead to a variety of abnormal conditions such as stress disorder, mitochondrial dysfunction, embryogenesis and neurodevelopmental abnormalities, and even tumor cell proliferation, migration, and tumorigenesis, etc. (Navarro et al., 2021; Walworth et al., 2021). Research in recent decades has found that multiple m5C methyltransferases are associated with some cancer phenotypes. Meanwhile, RNA m5C modification abnormalities have been reported to exert various functions in HCC tumorigenesis and progression (Du et al., 2020). A study used MeRIP-seq to analyze m5C methylation in HCC tissues and adjacent tissues, and the results showed a richer and higher m5C modification peak in the mRNA of cancer tissue than in adjacent tissues, which once again confirmed the role of m5C in HCC (Zhang Q. et al., 2020). Although the importance of m5C is highlighted, the study lacks in-depth mechanism exploration of the mechanism, so we have not found a potential target for HCC intervention. Therefore, clarifying the role and detailed mechanism of RNA m5C modification in HCC, screening and identifying potential therapeutic targets, and formulating promising targeted intervention strategies will further promote the treatment of HCC in the future.

To date, research on m5C in HCC has made some progress, as shown in Figure 2B. Many studies have shown that the high expression of almost all m5C regulators is significantly related to the shorter overall survival period of HCC patients except for NSUN7, suggesting that the dysfunction of m5C regulators has a strong impact on HCC progression (He et al., 2021). Since then, a study used clinical information from the TCGA database to assess m5C regulator alterations and the survival rate in patients and found that the *p*-value of the NSUN5 and ALYREF genes in HCC is less than 0.05, as well as the significant difference in the NSUN6 gene, which provides an important basis for exploring m5C regulators as potential therapeutic targets for HCC (Song et al., 2021). Moreover, gene set enrichment analysis (GSEA) showed that the high expression of NSUN4 and ALYREF mainly affects the m5C methylation/demethylation process, cell cycle regulation and mitosis, respectively, which is closely related to the prognosis of HCC (He et al., 2020a). In addition, the study found that the level of eukaryotic initiation factor 4A3 (eIF4A3) was significantly related to the expression of ALYREF, and the upregulation of eIF4A3 was prominently correlated with the poor prognosis of HCC patients. Furthermore, they found that eIF4A3 expression was significantly correlated with ALYREF expression and that upregulated eIF4A3 was significantly relevant to poor HCC patient outcomes. Protein–protein interaction network analysis identified 8 hub genes (PCNA, SNRPD1, MCM2, MCM3, RFC4, BIRC5, NOP56, and MCM6) based on the positive association between ALYREF and eIF4A3, and the high expression levels of these hub genes were positively associated with patient clinical outcomes (Xue et al., 2021). These findings indicated that the increased levels of NSUN4 and ALYREF may function as promising clinical biomarkers for both HCC diagnosis and prognosis, and in-depth research concentrating on NSUN4- and ALYREF-mediated m5C modification may provide novel therapeutic targets and targeted intervention strategies for HCC treatment. Functional studies have found that knocking out the RNA m5C transferase NSUN2 can significantly suppress the growth, angiogenesis, metastasis and invasion of HCC cells, and this result is closely related to the NUS2-mediated m5C methylation of H19 lncRNA (Sun et al., 2020). In detail, NUS2-mediated m5C modification can enhance the stability of H19 lncRNA, which will motivate the specific binding with the oncoprotein Ras GTPase-activating protein-binding protein 1 (G3BP1) and cause the overexpression of MYC, thus promoting the malignancy of liver cells and leading to the carcinogenesis of HCC. These results revealed that targeting the NSUN2-mediated m5C methylation of H19 lncRNA might be a new therapeutic strategy for HCC treatment.

In addition, some studies have also found thousands of m5C methylation peaks in circRNAs of HCC tissue, and the number and distribution of these methylation peaks are significant between HCC and adjacent tissues (He et al., 2020b). Whole chromosome level analysis showed a significantly different distribution of m5C-methylated circRNA in multiple chromosomes between cancer and adjacent tissues, especially the X chromosome, suggesting that m5C changes extensively in HCC tissues and may affect HCC phenotypes through multiple avenues (Torsin et al., 2021). Although research is still on the surface, the results remind us that circRNA m5C modification has researchable value in the carcinogenesis and development of HCC. In-depth studies of the mechanisms can be carried out to determine whether it can be regarded as a potential therapeutic target for HCC.

In a variety of physiological processes and diseases, studies have shown that m5C methylation not only regulates mRNA, lncRNA and circRNA to affect disease progression but also affects the functions of tRNA and rRNA. It seems that the current research on m5C in liver cancer is only the tip of the iceberg. If research continues, more m5C functions in liver cancer will be discovered. We look forward to in-depth exploration of the mechanisms to identify and illustrate new potential therapeutic targets based on m5C research, which will be a research breakthrough on targeted intervention strategies for liver cancer.

## RNA N1-Methyladenosine Epigenetic Modification in Hepatocellular Carcinoma

As early as the 1960s, researchers confirmed for the first time that m1A is a crucial RNA posttranscriptional modification in isolated rat liver RNA (Zhang and Jia, 2018). Although the m1A modification has been discovered for more than six decades, the role and mechanism of this RNA modification has not progressed until recently (Wiener and Schwartz, 2021). To date, a number of studies have confirmed that m1A is mainly produced by transferring a methyl group to the N1 position of adenosine, which can be regulated by various m1A methylation regulators, primarily including “writers” (TRMT6, TRMT61A, TRMT61B, and TRMT10C), “erasers” (ALKBH1 and ALKBH3), and “readers” (YTHDF1-3 and YTHDC1) (Liu et al., 2016; Safra et al., 2017).

The “writers” consist of a methyltransferase complex, which deposits the m1A label. TRMT61B is mainly located in the mitochondria and forms homogeneous oligomers, while TRMT61A can form α2β2 heterotetramers with TRMT6, which regulates the m1A methylation of cytoplasmic tRNA (Li X. et al., 2017). m1A demethylases eliminate the methyl group in m1A like “erasers” to make the function of m1A reversible. The function of “readers” is mainly to help decode m1A methylation to mediate the posttranscriptional downstream effects (Wei et al., 2018). Generally, “writers” and “erasers” determine the occurrence and distribution of m1A, whereas “readers” help promote m1A functions. m1A dysfunction affects a variety of biological processes, such as cell proliferation, self-renewal programs, and apoptosis (Wang, 2019). These processes not only maintain normal biological functions but also have key roles in carcinogenesis and cancer progression. A study showed that the high expression of the m1A transmethylase hTRM6P/hTRM61P is related to the urinary m1A level and bladder carcinogenesis (Shi et al., 2015). Moreover, abnormally expressed m1A-related regulatory genes are closely associated with the mTOR and ErbB pathways in gastrointestinal cancers (Zhao et al., 2019).

Currently, although research on m1A in HCC has progressed, it is not deep enough, as shown in Figure 2C. Some researchers used The Cancer Genome Atlas-Liver Hepatocellular Carcinoma (TCGALIHC) database to explore the relationship between 10 m1A regulators (TRMT6, TRMT61A, TRMT61B, TRMT10C, YTHDF1-3, YTHDC1, ALKBH1, ALKBH3) and the relevant clinicopathological characteristics, sequencing results, their relationship and the impact of genetic changes on survival; the results showed that high levels of TRMT6, TRMT61A, TRMT10C, and YTHDF1 are closely associated with the poor prognosis of HCC patients, with both AUC1y and AUC3y greater than 0.66 (Shi Q. et al., 2020). Subsequently, an authoritative independent validation dataset showed that these 4 genes have good risk prediction capabilities for HCC (*p* = 0.011, AUC > 0.67), while a high TRMT6 level was related to poor prognosis (*p* = 0.014) (Safra et al., 2017). These results indicate that the levels of these four m1A modifier genes in liver tissue have significant relevance to the clinical diagnosis and treatment of HCC patients and may become potential targets in HCC research and prevention. Subsequently, GSEA was used to study the potential biological functions of four m1A modifier genes in the pathogenesis of HCC, and the results showed that the increased expression of TRMT6 is related to multiple biological functions in the nucleus (Wang et al., 2019). Upregulated TRMT61A is involved in protein metabolism, while high TRMT10C expression is associated with the MYC pathway and cell division, and a high level of YTHDF1 is relevant to cell mitosis (Hartl, 2016). The aforementioned findings not only provide new clues and potential targets for the risk prediction, diagnosis and prognosis of HCC but also show the critical roles of m1A-related writers and readers in mediating biological processes in HCC. If in-depth *in vivo/vitro* studies can be conducted on this basis to clarify the mechanism of these regulators (TRMT6, TRMT61A, TRMT10C, and YTHDF1), they will provide promising targeted intervention strategies for HCC therapy. Meanwhile, a recent study showed that the expression levels of m1A erasers, including ALKBH1 and ALKBH3, in HCC were obviously higher than those in adjacent tissue (Hartl, 2016; Shi Q. et al., 2020). Specifically, the overexpression of ALKBH1 is negatively correlated with the overall survival rate of HCC, indicating that ALKBH1 may be regarded as a pivotal poor prognosis marker for HCC, but further research is needed (Ma et al., 2019). When assessing the correlation between m1A modifier genes and HCC tumor stage, it was found that high levels of TRMT6, TRMT61A, TRMT10C, ALKBH3, and YTHDF2 are significantly positively correlated with higher tumor stage (G1-G3), which also showed the good evaluation and predictive value of ALKBH3 in the typing and staging of HCC (Wang et al., 2018). The results of the aforementioned studies are highly consistent, indicating that m1A RNA epigenetic modification performs crucial functions in regulating the progression of human HCC.

m1A RNA epigenetic modification reveals the emerging role and potential research value of epigenetic regulation of gene expression in the occurrence and development of liver cancer. However, since the study of m1A in liver cancer is currently in its infancy compared with m5C and m6A, corresponding molecular mechanism research is extremely lacking. Therefore, in-depth molecular mechanism research can be carried out on the basis of clinical research to clarify the functions of m1A methylation in HCC, which will greatly promote the discovery of new targets for the treatment of liver cancer and the development of targeted intervention strategies.

## RNA N7-Methylguanosine Epigenetic Modifications in Hepatocellular Carcinoma

An initial study found that m7G mainly exists in the internal sites of rRNA and tRNA (Lin S. et al., 2019). However, previous detection methods, such as thin layer chromatography, capillary electrophoresis, and mass spectrometry, cannot distinguish between m7G on 5′-mRNA and internal m7G; it is impossible to determine whether m7G modification is present in mRNA (Enroth et al., 2019). With the deepening of research, m7G modification has been shown to have a wide range of effects on tRNA, rRNA, and mRNA and plays a pivotal role in many biological processes, such as transcription elongation, premRNA splicing, nuclear export, and mRNA translation (Zhang et al., 2019; Campeanu et al., 2021). In the process of RNA m7G modification, METTL1-WDR4, i.e., an m7G methyltransferase complex, mediates the m7G modification of cytoplasmic tRNAs and mRNA, which regulates premRNA splicing, RNA export, stability and translation in mammals, while the heterodimer Trm8-Trm82 plays the same role in yeast (Matsumoto et al., 2008; Dai et al., 2021). Meanwhile, Williams-Beuren syndrome chromosome region 22 (WBSCR22) mediates the 18S rRNA m7G modification at position 1639, thus regulating the processing and maturation of rRNA and biosynthesis of the 40S ribosomal subunit in the nucleus (Haag et al., 2015; Zorbas et al., 2015). Studies have found that the internal m7G methylation of these RNAs not only plays certain roles in multiple biological processes, such as RNA processing and maturation, but is also closely related to several human diseases (Haag et al., 2015; Zorbas et al., 2015; Katsara and Schneider, 2021; Ma et al., 2021). For instance, METTL1-WDR4 complex mutations may lead to a unique form of microcephalic primitive dwarfism (Shaheen et al., 2015; Lin et al., 2018). A recent study reported that METTL1 can promote miRNA let-7e processing in an m7G modification-dependent manner, which is involved in the regulation of cancer progression (Shaheen et al., 2015; Lin et al., 2018; Pandolfini et al., 2019; Liu et al., 2020). Meanwhile, a study found that the main components of the m7G methyltransferase complex, namely, METTL1 and WRD4, were significantly upregulated in intrahepatic cholangiocarcinoma tissues compared with adjacent tissues and had a strong correlation with poor prognosis (Orellana et al., 2021). In-depth examination indicated that m7G methyltransferase-mediated tRNA modification can selectively regulate oncogenic transcript translation *via* m7G-tRNA-decoded codon-frequency-dependent mechanisms, including the epidermal growth factor receptor (EGFR) pathway and cell cycle-related genes (Dai et al., 2021). These results suggest that m7G modification plays a decisive role in many diseases, including cancers, and requires great attention and in-depth research.

Although research on HCC is not as abundant as that on other tumors, it finally allows us to see the exact role of m7G, as shown in Figure 2D. Researchers used the genome-wide atlas to explore promising therapeutic targets of HCC and found that the levels of WBSCR22 and the other five genes (EXOSC4, RNMT, SENP6, RASAL2, and NENF) were much higher in HCC tissues than in adjacent tissues (Li C. et al., 2017). Moreover, silencing WBSCR22 in several human liver cancer cell lines can obviously suppress cell growth and invasive properties (Stefanska et al., 2014). The aforementioned research indicated that as an important RNA m7G modification regulator, WBSCR22 plays a significant role in the growth and invasion of HCC cells and can be considered a potential therapeutic target for HCC in-depth research. Regrettably, the relevant research has not continued in depth, but it also offers novel clues and theoretical supports for research on the role of m7G methylation in the occurrence and development of liver cancer. Subsequently, a study began to investigate the clinical significance and potential value of the m7G methyltransferase WDR4 in liver cancer. A study found that the high expression of WRD4 in HCC tissues can significantly increase the level of m7G methylation and is related to the poor prognosis of HCC patients (Xia et al., 2021). Mechanistically, c-MYC can activate the transcription of WDR4, and then, activated WDR4 enhances the stability of cyclin B1 (CCNB1) mRNA and promotes its translation through specific tRNA m7G methylation. Subsequently, CCNB1 increases the ubiquitination of p53 to increase the phosphorylation of PI3K and Akt and reduces the expression of p53 protein, which ultimately facilitates the proliferation of HCC cells by inducing G2/M cell cycle transition, inhibiting apoptosis, and enhancing metastasis and sorafenib resistance *via* EMT (Liu J. S. et al., 2019). The above results indicated that the RNA m7G methyltransferase WDR4 acts as a tumor promoter during the carcinogenesis and progression of HCC and may be regarded as a promising therapeutic target for HCC treatment. In a clinical study covering 892 patients with liver cancer, abnormally high METTL1 expression was significantly positively correlated with serum AFP level, tumor volume, tumor vascular infiltration, and poor prognosis. In addition, multivariate analysis showed that METTL1 is an independent factor affecting overall survival (Dai et al., 2021). Mechanistically speaking, overexpression of METTL1 promotes the proliferation and migration of liver cancer cells, while knockout of METTL1 leads to the opposite phenotype, possibly because METTL1-mediated RNA m7G methylation inhibits phosphatase and tensin homolog (PTEN) signal transduction. Relevant experiments have confirmed that the ectopic expression of PTEN or the inhibition of Akt activity can significantly reduce the malignant phenotype mediated by METTL1 (Tian et al., 2019). In summary, the methylation modification of RNA m7G mediated by the methyltransferase METTL1 plays an indispensable role in the growth, invasion and malignant phenotypic transformation of liver cancer cells. METTL1 is a promising diagnostic and prognostic biomarker in HCC, and targeting METTL1-mediated RNA m7G methylation will provide hope for HCC intervention.

Generally, people have recognized the important role of RNA m7G modification in a series of biological processes and disease progression, while its role and detailed mechanism in the occurrence and development of HCC have not yet been elucidated. Although the methyltransferases METTL1, WRD4 and WBSCR22 are closely related to the diagnosis and poor prognosis of HCC, more research is needed to realize the promotion of m7G methyltransferase as a potential therapeutic target and the positioning of RNA m7G methylation to develop a promising targeted intervention strategy.

## Other RNA Epigenetic Modification Processes in Hepatocellular Carcinoma

In addition to the aforementioned RNA methylation modifications, the C5-glycoside isomer pseudouridine (ψ) of uridine is the first posttranscriptional modification discovered and one of the most abundant modifications in RNA (Cui et al., 2021). Pseudouridine was originally found on yeast tRNAs and rRNA, and recent research has pointed out that ψ modification was also found in other types of RNA, including miRNAs, lncRNAs, small nuclear RNAs (snRNAs), and small Cajal body-specific RNAs (scaRNAs) (Gilbert et al., 2016; Yamaki et al., 2020). At present, a total of 13 types of pseudouridine synthases (PUSs) have been discovered, including dyskerin PUS1 (DKC1), PUS1, PUS3, PUS7, PUS10, PUS1-like (PUSL1), PUS7L, RNA PSU domain-covering 1 (RPUSD1), RPUSD2-4, TruB PSU class members 1 (TRUB1), and TRUB2 (Rintala-Dempsey and Kothe, 2017). Studies have shown that pseudouridine can be achieved by two different mechanisms: RNA-independent and RNA-dependent pseudouridine. RNA-independent pseudouridine is catalyzed by an enzyme, namely, PUSs, which perform substrate recognition and catalysis without RNA template strands, while the RNA-dependent mechanism is mainly achieved *via* RNA-protein complexes, also called Box H/ACA small ribonucleoproteins (snoRNPs) (Kim et al., 2021). Studies have pointed out that pseudouridylation plays a specific role in different aspects of gene expression and regulation, which is closely related to the RNA type being modified (Kierzek et al., 2014; Zhao and He, 2015). Specifically, pseudouridines were found at the tRNA binding site, ribosomal subunit interface, peptidyl transferase center, decoding site and mRNA channel, which contributed to the correct assembly and function of ribosomes and protein synthesis in rRNA (Penzo and Montanaro, 2018). In tRNAs, pseudouridylation mainly acts on conserved position 55 of the anticodon stem and loop, D stem and ψ loop to stabilize the tertiary structure of tRNAs, thereby promoting codon-anticodon base pairing (Guzzi et al., 2018). In mRNA, ψ mainly facilitates non-sense-to-sense codon conversion and assists base pairing at the decoding center of the ribosome, which leads to protein diversity (Carlile et al., 2019). In addition to participating in diverse biological processes, ψ also affects the progression of diseases. One of the common pseudouria deficiency diseases is X-linked congenital keratosis (X-DC), which is closely related to the mutation and inactivation of DKC1 (Thumati et al., 2013). In addition, the role of PUS10 is indelible during TNF-related apoptosis, inducing ligand (TRAIL)-induced prostate cancer cell apoptosis, and changes in its locus in the genome are significantly involved in the risk of lung cancer (Stockert et al., 2019, 2021).

In a study of HCC (Figure 2E), it was found that DKC1 was significantly upregulated in human cancer tissue and was significantly positively correlated with the cancer cell proliferation potential, advanced clinical stage and prognosis of HCC patients (Liu et al., 2012). Despite the lack of in-depth studies, it has provided a new direction and background reference for elucidating the role and mechanism of hepatocarcinogenesis and progression. Recent studies have reported that abnormally expressed snoRNA-mediated pseudouridylation at the U609 and U863 sites of the rRNA 18S subunit leads to abnormal tRNA selection efficiency, ribosome elongation rate and translation efficiency, thus affecting HCC cell survival; however, the role of DKC1 in the whole process cannot be ignored (McMahon et al., 2019; Nombela et al., 2021). From a clinical perspective, ψ or its regulators, such as DKC1 and PUSs, may become potential biomarkers and therapeutic targets in cancer treatment. However, the role of mutations and expression changes of DKC1 in HCC has been clarified, and the effects of other synthetases related to pseudoouridine are rarely reported. With the deepening of cancer gene transcriptome research and the development of computational analysis, the role of pseudouridine-related enzymes in the initiation, development, metastasis and resistance of HCC will be investigated clearly, which will provide an essential reference for the strategy of targeting pseudouridine in the treatment of HCC.

Furthermore, we expect that technological development can help us identify the more important roles of RNA epigenetic modification in the carcinogenesis and progression of HCC to provide novel therapeutic targets and targeted intervention strategies for the research, prevention and treatment of HCC.

## Perspectives of Targeted Intervention Strategies

RNA epigenetic modification has become a key means of posttranscriptional regulation in the process of gene expression. Based on the critical role of RNA-modified regulatory proteins in the carcinogenesis, maintenance, invasion and metastasis of HCC, especially as valuable clinical diagnosis and poor prognosis markers, they are promising therapeutic targets because their activity can be interfered with by multiple mechanisms, such as gene editing and small molecules (Juhling et al., 2021). Although reliable intervention strategies and targeted drugs based on RNA epigenetic modification for HCC have not yet been established, there have been some successful cases in other tumors, such as myeloid leukemia and glioblastoma, which provide hope and a reference for HCC-related research (Bayo et al., 2019).

A study found that the m6A demethylase FTO was inhibited by the oncometabolite R-2-hydroxyglutarate (R-2HG), leading to increased methylation of acute myeloid leukemia (AML) and glioma cells and reduced mRNA expression of c-MYC and CEBPA, thereby blocking cell proliferation and the cell cycle and inducing apoptosis, which play a therapeutic role in AML and glioma (Qing et al., 2021). Since then, other studies have attempted to develop small molecule inhibitors against the RNA demethylases AKLBH5 and FTO, giving promising results at the preclinical stage. For example, the FDA-approved ethyl ester form of meclofenamic acid (MA), a non-steroidal anti-inflammatory drug named MA2, was found to be an FTO inhibitor that increased the m6A modification level of mRNA in glioblastoma cells, thus inhibiting tumor progression and extending the lifespan of GSC-transplanted mice (Xiao et al., 2020). Meanwhile, some research groups have designed 2-oxoglutarate and iron-dependent oxygenase (2OGX) inhibitors to target m6A erasure agents, such as IOX3 inhibitors, based on the structure of the FTO and ALKBH5 domains (Aik et al., 2014). However, these promising inhibitors need to carefully consider their limitations before clinical use. The latest research has developed a highly efficient and selective first-in-class catalytic inhibitor, STM2457, for METTL3 and the identification and characterization of its cocrystal structure combined with the m6A methyltransferase complex METTL3/14 (Yankova et al., 2021). This inhibitor not only significantly inhibits the growth, differentiation and apoptosis of AML caused by METTL3 but also selectively decreases the m6A level of leukemia-related mRNA; the decrease in its expression is consistent with translation defects.

The *in vivo* inhibition of METTL3 results in impaired engraftment and prolongs survival in multiple mouse models of AML, especially the key stem cell subpopulations of AML. In general, this study reveals that inhibiting METTL3 pharmacologically can be used as a potential therapeutic strategy for AML and provides a novel concept for HCC treatment by targeting m6A-modified enzymes. When targeting other RNA epigenetic modification processes and corresponding regulatory proteins, a study found that using azacytidine to completely inhibit DNMT2-mediated tRNA m5C methylation can significantly reduce cancer cell proliferation, which supports the idea that reducing tRNA m5C methylation may be an effective cancer therapeutic strategy (Cheng et al., 2018). Although these results have brought new light to cancer treatment, these analogs may have devastating consequences because they can affect unique targets in different cells, organelles and genes (DNA, tRNA, rRNA, mRNA, ncRNA). Consistent with the view that inhibiting the methylase of tRNA may lead to chemotherapy resistance, silencing other tRNA methyltransferases, such as m7G methylase METTL1, will undergo methylation modification on several tRNA variable loops, which can enhance the sensitivity of cancer cells to 5-FU (Okamoto et al., 2014).

In terms of drug design or small molecule screening for targeted inhibition of PUSs’ activities, although studies have synthesized or screened compounds that can inhibit DKC1 as potential targeted anticancer therapies, they have found little effect in clinical trials (Schwartz et al., 2014). Pyrazofurin is a small molecule inhibitor of orotodine 5′-monophosphate decarboxylase (ODCase), which inhibits the activity of DKC1 (Ren et al., 2019). To test the effectiveness of pyrazofurin as an anticancer drug, a number of clinical trials have been conducted in ovarian cancer, sarcoma, colorectal cancer, AML, breast cancer, lung cancer, melanoma, and other cancers. In all cases, pyrazofurin showed no efficient anticancer activity. However, since the expression level of DKC1 is not considered, it is unknown whether pyrazofurin can treat cancer patients with DKC1 overexpression (Kan et al., 2021). Floresta et al. (2018) hypothesized and discovered that nucleoside analogs such as isoxazolidinyl derivative 5′-monophosphate have higher ligand efficiency in the enzyme active site than the natural substrate. It can be used as an inhibitor of pseudouracil 5′-monophosphate glycosidase to compete with natural substrates to prevent the cleavage of glycoside C-C bonds (Floresta et al., 2018). Although they ignore the tumor growth inhibitory potential and the therapeutic benefits of using these inhibitors, these studies laid the foundation for the continued search for ψ synthase inhibitors for cancer treatment.

The above research results show that targeted RNA epigenetic modification, corresponding regulatory protein screening, and targeted intervention strategy exploration are promising research directions. However, inhibitors, drugs and targeted intervention strategies based on these RNA epigenetic modifications and regulators have not made ideal progress in HCC treatment. From the perspective of pharmacology, it is necessary to explore effective, highly selective inhibitors or analogs with ideal biological activity to determine the therapeutic benefits and potential risks of targeted interventions with these regulators if these modifications and corresponding modulators are to be validated as promising pharmacological targets. Therefore, it is necessary to design and optimize targeted intervention strategies for targeting RNA epigenetic modification and its regulatory proteins for the treatment of HCC to realize the therapeutic potential of regulating RNA epigenetic modification in HCC and other diseases.

## Conclusion

The main reason for the high mortality/poor prognosis of HCC is the malignant growth, invasion, metastasis and refractoriness of the tumor (Ioannou, 2021). Despite the ongoing development of medical technology and the increasing abundance of treatment methods, HCC is still one of the urgent problems to be solved in the era of precision medicine due to the unexplained molecular mechanism of its carcinogenesis and development, lack of ideal therapeutic targets and targeted intervention strategies (Jiri et al., 2020). In continuous basic and clinical explorations, different RNA epigenetic modifications mediated by multiple regulators dynamically and reversibly regulate HCC cell proliferation and metabolism, providing new directions and ideas for the screening of potential therapeutic targets and the study of precise targeted intervention strategies for future HCC treatment.

## Author Contributions

W-JN and HZ designed the “idea” and revised the manuscript. L-RZ, W-JN, and MC wrote the manuscript. W-JN and W-TD collected the information. All authors read and approved the final manuscript.

## Conflict of Interest

The authors declare that the research was conducted in the absence of any commercial or financial relationships that could be construed as a potential conflict of interest.

## Publisher’s Note

All claims expressed in this article are solely those of the authors and do not necessarily represent those of their affiliated organizations, or those of the publisher, the editors and the reviewers. Any product that may be evaluated in this article, or claim that may be made by its manufacturer, is not guaranteed or endorsed by the publisher.
